# Neural substrates for anticipation and consumption of social and monetary incentives in depression

**DOI:** 10.1093/scan/nsz061

**Published:** 2019-09-11

**Authors:** Zhenhong He, Dandan Zhang, Nils Muhlert, Rebecca Elliott

**Affiliations:** 1 School of Psychology, Shenzhen University, 518060 Shenzhen, China; 2 Division of Neuroscience and Experimental Psychology, School of Biological Science, University of Manchester, M13 9PL Manchester, UK and; 3 Shenzhen Key Laboratory of Affective and Social Cognitive Science, Shenzhen University, 518060 Shenzhen, China

**Keywords:** social incentive, depression, anticipation, subgenual cingulate cortex, insula, ventral lateral pre-frontal cortex

## Abstract

Depression has been reliably associated with abnormalities in the neural representation of reward and loss. However, most studies have focused on monetary incentives; fewer studies have considered neural representation of social incentives. A direct comparison of non-social and social incentives within the same study would establish whether responses to the different incentives are differentially affected in depression. The functional magnetic resonance imaging study presented here investigated the neural activity of individuals with subthreshold depression (SD) and healthy controls (HCs) while they participated in an incentive delay task offering two types of reward (monetary gain *vs* social approval) and loss (monetary loss *vs* social disapproval). Compared to HCs, individuals with SD showed increased subgenual anterior cingulate cortex (sgACC) activity during anticipation of social loss, whereas the response in the putamen was decreased during consumption of social gain. Individuals with SD also exhibited diminished insula responses in consuming social loss. Furthermore, positive connectivity between the insula and ventral lateral pre-frontal cortex (VLPFC) was observed in individuals with SD while negative connectivity was found in HCs when consuming social loss. These results demonstrate neural alterations in individuals with depression, specific to the processing of social incentives, mainly characterised by dysfunction within the ‘social pain network’ (sgACC, insula and VLPFC).

## Introduction

Depression is characterised by altered processing of reward and loss (Eshel and Roiser, 2010; McCabe *et al*., 2012). Compared to healthy controls, depressed individuals show a maladaptive response to punishment, i.e. hypersensitivity to punishment or failed use of negative feedback to improve their future performance, combined with hyposensitivity to reward (Eshel and Roiser, 2010). This abnormality to reward and loss has been linked to aberrant function of frontal [dorsolateral and medial pre-frontal cortex (DLPFC, MPFC)] and ventral striatal regions (nucleus accumbens, putamen and caudate; Forbes *et al*., 2009; Knutson *et al*., 2008; Pizzagalli *et al*., 2009). While neural mechanisms of these abnormalities have been extensively explored for incentives such as money and food (Rizvi *et al*., 2016), another important incentive, i.e. social feedback (e.g. praise or conversely, disapproval), has been largely ignored (Spreckelmeyer *et al*., 2009). It is increasingly recognized that, compared to non-social feedback, social feedback processing is more likely to be affected by depression (Forbes, 2009; Brinkmann *et al*., 2014). There is therefore a clear rationale for exploring social feedback processing in depression.

Depressed individuals show blunted response to social rewards, decreased enjoyment and reduced interest in social activity (i.e. social anhedonia; for a review, see Kupferberg *et al*., 2016), while they are hypersensitive to social losses like social rejection (Hsu *et al*., 2015). Although impairments of non-social and social processing are consistent in depression at the behavioural level, i.e. hypersensitive to punishment and insensitive to rewarding (Eshel and Roiser, 2010; Kupferberg *et al*., 2016), previous neuroimaging studies have revealed that the neural manifestation between monetary and social incentives is somewhat different in depression. For instance, it is found that while depressed individuals show decreased striatal response to monetary reward (Forbes *et al*., 2006), they exhibit increased amygdala response to social reward (social acceptance; Davey *et al*., 2011). Furthermore, depressed individuals have greater activation for social punishment in brain areas including the ventral lateral pre-frontal cortex (VLPFC), insula and subgenual anterior cingulate cortex (sgACC), all regions of the ‘social pain’ network (Silk *et al*., 2014; Rudolph *et al*., 2016; Kumar *et al*., 2017; Jankowski *et al*., 2018). In contrast, reduced reactivity across multiple brain regions including pre-frontal regions, parietal cortex, amygdala and putamen have been observed for monetary losses in individuals with depression (Knutson *et al*., 2008; Schiller *et al*., 2013).

The processing of reward and loss can be divided into two distinct phases, i.e. consummatory and appetitive phases (Berridge, 1999; Rademacher *et al*., 2010). The monetary incentive delay (MID) paradigm and its social version, i.e. social incentive delay (SID) paradigm allow for separate investigation of the two phases so to distinguish between appetitive process during anticipation and hedonic process during consumption of reward and loss (Knutson *et al*., 2000; Spreckelmeyer *et al*., 2009). Using the MID and similar paradigms, evidence suggests that anhedonia, a core symptom in depression, can be observed in not only the ‘liking’ but also the ‘wanting’ phase (Pizzagalli, 2014; Rizvi *et al*., 2016). While reduced fronto-striatal activity during monetary reward anticipation has been suggested as the major neural correlate of an abnormal ‘wanting’ process in depression (Knutson *et al*., 2008; Pizzagalli *et al*., 2009; Smoski *et al*., 2009; Ubl *et al*., 2015), dysfunction in other brain regions has also been reported, e.g. increased dorsal ACC activation is usually considered to reflect increased conflict during anticipation of monetary gains (Knutson *et al*., 2008).

However, while there is emerging research on anticipation of monetary incentives, relatively little is known about the neural basis of anticipation of social incentives in depression. Behavioural studies have indicated that individuals with depression reported more expectation of negative and less expectation of positive social feedback (i.e. negative expectancy bias) compared to control participants (Feeser *et al*., 2013; Caouette and Guyer, 2016; Setterfield *et al*., 2016). Thus, behavioural evidence suggests altered anticipatory processing in individuals with depression, but the neural basis of social incentive anticipation in individuals with depression has yet to be studied. Furthermore, since there are few studies presenting non-social and social incentives within the same experiment (Rademacher *et al*., 2010; Hames *et al*., 2013), it is unclear whether there is specific and dissociable neural deficits for social feedback processing in depression.

To address the issue, the current study therefore aimed to directly compare the neural correlates of monetary and social incentive processing in individuals with subthreshold depression (SD), with the hypothesis that SD is associated with neural dysfunctions in anticipating and experiencing monetary and (particularly) social incentives. We focused on SD as it allows for investigating potential depression vulnerability indices and excludes the influence of antidepressant medications or other clinical treatments on the incentive processing. We combined the MID and SID tasks in which neural responses could be examined using two types of reward (monetary gain and social approval) and loss (monetary loss and social disapproval). In healthy people, evidence suggest that non-social (e.g. money) and social incentive processing overlap in their underlying neural substrates (Izuma *et al*., 2008; Spreckelmeyer *et al*., 2009; Lin *et al*., 2012; Gu *et al*., 2019). For instance, neuroimaging meta-analysis and relevant studies revealed robust activation in striatum, ACC, insula and VLPFC in tasks presenting monetary or social incentives (Spreckelmeyer *et al*., 2009; Wilson *et al*., 2018). Individuals with depression have reduced striatum and increased ACC activity in response to monetary incentives (Knutson *et al*., 2008; Pizzagalli *et al*., 2009), while they show increased ACC, insula and VLPFC responses in processing social incentives (Silk *et al*., 2014; Kumar *et al*., 2017; Jankowski *et al*., 2018). Therefore, we expected that in individuals with SD, processing both types of incentive would share a common pattern of increased ACC response, while reduced striatum would be specific to the processing of monetary incentives, and increased VLPFC and insula would be specific to social incentive processing.

## Methods

### Participants

In a mental health screening of Shenzhen University, the Self-rating Depression Scale (SDS, Zung *et al*., 1965), the Beck Depression Inventory Second Edition (BDI-II, Beck *et al*., 1996) and the Trait form of Spielberger’s State-trait Anxiety Inventory (STAI-T, Spielberger *et al*., 1983) were administered to all the freshman students (approximately 6000 students). This study included individuals from this sample with SD indexed by (1) level of depressive severity (measured by SDS) > 0.5 and (2) BDI-II scores > 13. Note: according to the norms of SDS (Zung *et al*., 1965), a SDS score > 0.5/0.6/0.7 indicates mild, moderate and severe depression; according to the norms of BDI-II (Zung *et al*., 1965), a BDI-II score > 13/19/28 indicates mild, moderate and severe depression.

Exclusion criteria included: (i) any lifetime Axis I disorders other than depression according to Structured Clinical Interview for DSM-IV-TR Axis I Disorders, Research Version, Non-patient Edition (SCID-I/NP; First *et al*., 2002); (ii) high level of anxiety, i.e. students with STAI-T scores ranked above 75% of the distribution (Xie *et al*., 2018); (iii) seizure disorder; (iv) history of head injury with possible neurological sequelae; (v) self-reported prior use of any psychoactive drugs especially medication for depression; and (vi) current alcohol drug dependence.

Age- and gender-matched healthy controls (HCs) were recruited from the same sample source as individuals with SD. These participants had scores of depressive severity <0.5 (measured by SDS) and satisfied the same exclusion criteria as individuals with SD; furthermore, the HCs were screened with SCID-I/NP to guarantee without depression. Among the students who met the above criteria, 45 individuals (23 individuals with SD and 22 HCs) participated in the current study. Written informed consent was obtained prior to the experiment. The study was approved by the Ethics Committee of Shenzhen University.

Four participants failed to complete the experiment due to technical problems or personal discomfort, so the data from 41 individuals (20 females; 19 ± 1.7 years old, mean ± s.d.) were included for data analysis. As shown in Table 1, no significant difference was found between the two groups with respect to gender (χ^2^ = 0.161, *P* = 0.217), age, handedness and STAI-T scores.

**Table 1:** Demographic characteristics of the participants (mean and s.d.). SDS, Self-rating Depression Scale; STAI-T, the Trait form of Spielberger’s State-trait Anxiety Inventory; BIS/BAS, Behavioural Inhibition/Avoidance Scales; SHAPS, the Snaith–Hamilton Pleasure Scale; SPSRQ, the Sensitivity to Punishment and Sensitivity to Reward Questionnaire. Independent samples *t*-test was performed (two-tailed)

Items	SDs (*n* = 21)	HCs (*n* = 20)	*t*	*P*
Gender (male/female)	13/8	8/12		
Age (years)	19.76 (1.92)	19.45 (1.57)	0.57	0.574
Handedness, right/left	21/0	20/0		
SDS	0.54 (0.04)	0.42 (0.06)	7.49	<0.001
BDI-II	16.00 (8.00)	7.80 (5.79)	3.74	0.001
STAI-T	46.67 (6.22)	44.40 (4.37)	1.34	0.187
BIS/BAS				
BIS	14.10 (2.96)	15.60 (3.39)	−1.51	0.138
BAS Drive	8.48 (2.20)	7.30 (2.15)	1.73	0.092
BAS Fun Seeking	8.00 (1.84)	8.00 (1.59)	<0.01	1.000
BAS Reward Responsiveness	8.62 (1.72)	8.25 (1.80)	0.67	0.506
SHAPS	1.38 (1.75)	0.55 (0.89)	1.94	0.063
SPSRQ				
Sensitivity to reward	34.43 (4.58)	35.10 (2.94)	−0.56	0.581
Sensitivity to punishment	32.90 (4.95)	35.00 (4.17)	−1.46	0.152

### Brief introduction of self-reported measures

Both the Self-rating Depression Scale (SDS; Zung *et al*., 1965) and BDI-II (Beck *et al*., 1996) are widely used questionnaires to assess depressive symptoms. The score of depressive severity measured by the SDS ranges from 0.25 to 1.0, with higher scores corresponding to higher depressive severity. The BDI-II scores from 0 to 63, with high scores indicating a high level of depressive tendency.

The STAI-T (Spielberger *et al*., 1983) scores from 20 to 80, with high scores corresponding to high levels of anxiety.

The Behavioural Inhibition/Activation Scale (BIS/BAS) is used to assess motivation of behavioural inhibition and behavioural approach (Carver and White, 1994). It contains four subscales: BIS, BAS-Drive, BAS-Fun Seeking and BAS-Reward Responsiveness. A high score on BIS or BAS indicates a greater tendency to regulate aversive or appetitive motives so as to avoid unpleasant, or to approach desired, events and stimuli.

The Snaith–Hamilton Pleasure Scale (SHAPS) is used to capture hedonic capacity (Snaith *et al*., 1995). It scores from 0 to 14. A higher SHAPS score indicates higher levels of anhedonia.

The Sensitivity to Punishment and Sensitivity to Reward Questionnaire (SPSRQ) is used to assess BIS and BAS functioning, respectively (Torrubia *et al*., 2001). It includes two subscales: sensitivity to punishment and sensitivity to reward. Higher scores indicate higher levels of sensitivity.

These measures of the two experimental groups are reported in Table 1.

### Procedure

Before the experiment, participants were required to complete the six questionnaires mentioned above. Prior to entering the functional magnetic resonance imaging (fMRI) scanner, participants performed a practice session consisting of 36 trials of both MID and SID tasks to familiarise themselves with the experiment. Then, the threshold of visual reaction time was assessed by a simple reaction time task for each participant to personalise the difficulty of the formal experiment.

**Fig. 1:**
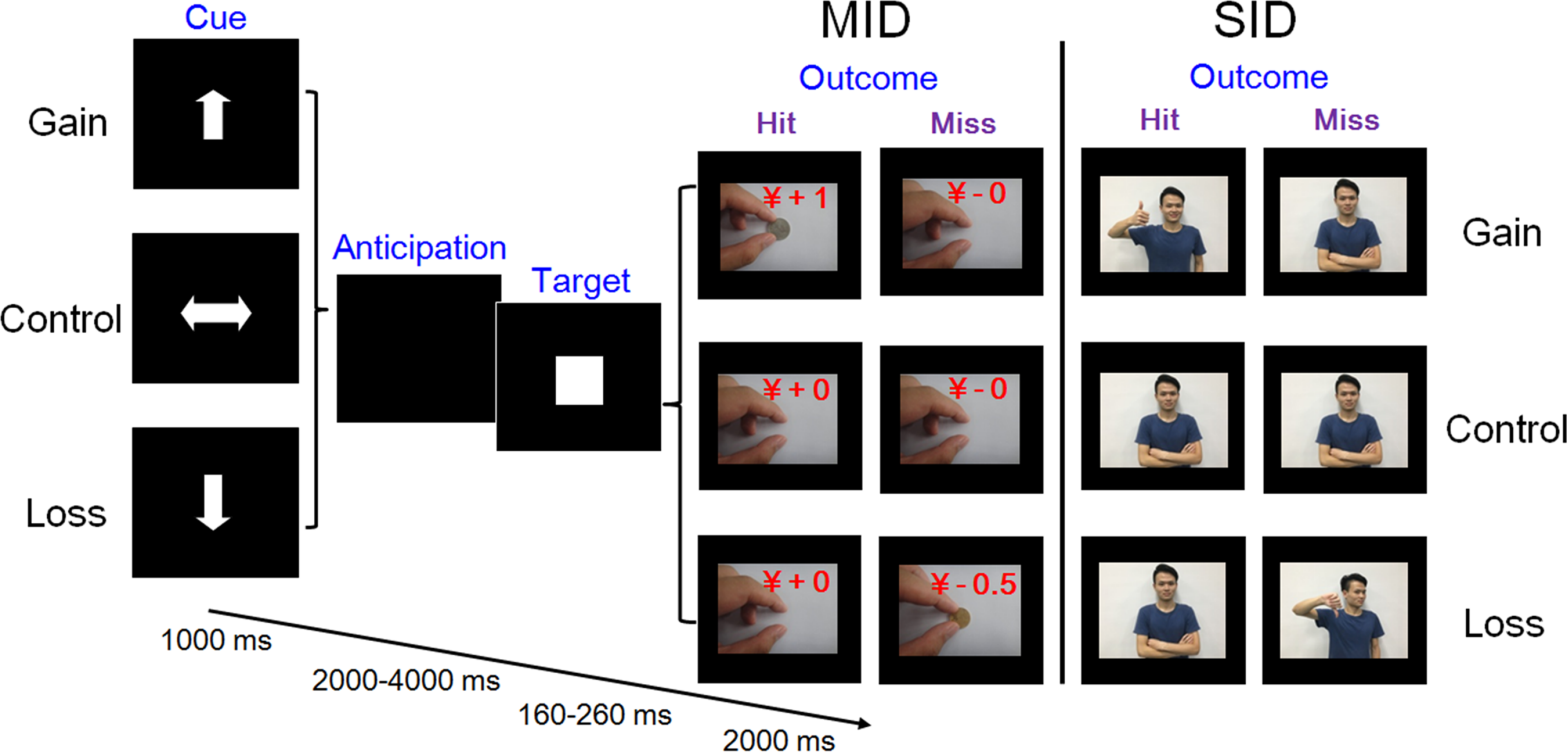
Schematic of trial sequence for the Monetary (MID) and the Social Incentive Delay (SID) Task. To increase the ecological validity of the paradigm, static photos were replaced with short movie clips during the outcome period. The person in the picture gave his consent for the material to appear in academic journals.

The scanning task comprised 2 MID and 2 SID blocks (randomly presented; see Figure 1), preceded by an instruction screen. In both MID and SID blocks, trials began with a cue indicating potential gains (an upward arrow), losses (a downward arrow) or ‘non-rewarded control’ (a horizontally oriented arrow) for 1 s, followed by a delay (anticipation) period for a various duration ranging from 2 to 4 s. After that, a white square (target) was presented; this indicated that participants had to press a button as quickly as possible in order to gain reward or avoid loss. The presentation time of the target was individually adjusted (between 160 and 260 ms) based on the pre-assessed threshold of visual reaction time. After the response, participants received visual feedback of short video clips for 2 s.

For the MID block, successful target hits in gain trials were followed by a short video clip showing a hand putting 1 Yuan on the desk while feedback for target misses was a neutral video clip showing the same hand putting nothing on the desk. Similarly in loss trials, feedback for misses was a video showing the hand removing 0.5 Yuan from the desk, while target hits were followed by the neutral video clip (Figure 1). The feedback video clips for SID blocks were analogous to those in MID blocks: successful target hits in gain trials resulted in a video clip showing a person who nodded with a smile and held up his/her thumb, while feedback for misses was a neutral video showing the same person with neutral facial expressions and body gestures. In loss trials, feedback for hits was the neutral clip while feedback for misses was a video clip showing a person who shook his/her head with a contemptuous facial expression and gave a ‘thumbs down’ gesture. In both MID and SID blocks, feedback in control trials was always the neutral video clip. There were four (paper and coin money with front or the other side) and eight (four actors and four actresses) sets of video clips for MID and SID blocks.

Participants were encouraged to respond to maximise their total rewards: for the MID block, their monetary gain and loss were linked to their final payment; for the SID block, they were told that their performance would be judged by peers assessing their IQ (with the assumption that faster reaction time is associated with higher IQ). Participants were led to believe that the ‘peers’ giving the feedback were the ones making the judging. Unbeknownst to participants, these ‘peers’ had not judged participants’ response capability, and the video clips presented during the scanning session were simply of volunteers who provided consent for their video to be used for scientific purposes. After the task, participants were asked if they really believed that their IQ was evaluated by the peers during the task. All the 41 individuals believed the cover story.

### Image acquisition

Brain images were collected using a 3 T Siemens TRIO MR scanner. Functional images were collected using an echo planar imaging sequence (number of slices, 41; gap, 0.6 mm; slice thickness, 3.0 mm; repetition time (TR), 2000 ms; echo time (TE), 25 ms; flip angle, 90°; voxel size, 3 mm × 3 mm × 3 mm; field of view (FOV), 200 mm × 200 mm). Structural images were acquired through 3D sagittal T1-weighted magnetisation-prepared rapid gradient echo (192 slices; TR, 2530 ms; TE, 3.39 ms; voxel size, 1.0 mm × 1.0 mm × 1.0 mm; flip angle, 7°; inversion time, 1100 ms; FOV, 256 mm × 256 mm).

### Image analysis

#### fMRI data processing

Images were pre-processed and analysed using Statistical Parametric Mapping (SPM8; http://www.fil.ion.ucl.ac.uk/spm). The first five volumes were discarded because of signal equilibration and participants’ adaptation to scanning noise. All remaining images were slice timing–corrected and realigned for motion correction by registration to the mean image. Artefact detection was conducted using the Artifact Detection Tools (ART) toolbox (https://www.nitrc.org/projects/artifact_detect); global mean intensity (>2 s.d. from mean image intensity for the entire scan) and motion (>2 mm) outliers were identified and entered as a regressor of no interest in the first-level general linear model (GLM) (Stoodley *et al*., 2012). Then, functional images were co-registered with the T1-weighted 3D images, normalized to standard MNI space and smoothed with a 6 mm FWHM isotropic Gaussian kernel. We chose this parameter as it was double the voxel size (3 mm) and would retain resolution for identifying changes in the relatively small brain regions we are interested in (see Pizzagalli *et al*., 2009; Ubl *et al*., 2015).

Pre-processed data were analysed as an event-related design in the context of the GLM approach in a two-level procedure. At the first level, regressors including three anticipation conditions (anticipation of gain, loss and neutral outcome) and three outcome conditions (feedback of gain, loss and neutral outcome) were modelled (a total of six factors). Due to the insufficient number of ‘miss’ trials in the behavioural task, only ‘hit’ trials during the outcome period were included for fMRI data analysis. Anticipation was the delay between cue disappearance and target appearance and was modelled as a brief block corresponding to the actual delay. Outcome was the feedback appearance, which was modelled as a single event with 0 s duration convolved with the canonical hemodynamic response function (HRF). To account for variance caused by head movement, six realignment motion parameters (three translations/rotations), and outlier scans identified by the ART toolbox were included as nuisance regressors in the model. Each normalized image was then high-pass filtered using a cutoff time constant of 128 s. In two tasks (MID, SID), contrast images for gain and loss were separately calculated for both anticipation and outcome stages, including (i) monetary gain *vs* monetary control, (ii) monetary loss *vs* monetary control, (iii) social gain *vs* social control, (iv) social loss *vs* social control, (v) (monetary and social) gain *vs* control, (vi) (monetary and social) loss *vs* control, (vii) monetary gain *vs* social gain, (viii) social gain *vs* monetary gain, (ix) monetary loss *vs* social loss and (x) social loss *vs* monetary loss.

These contrast images were taken to the second-level analysis. First, we performed one-sample *t*-tests, in which whole-brain analyses were computed for all contrasts separately for individuals with SD and HCs to identify whether the paradigm had activated brain regions as established in previous studies (Silk *et al*., 2014; Kumar *et al*., 2017; Jankowski *et al*., 2018; Wilson *et al*., 2018). To detect group differences, two-sample *t*-tests were also conducted at the whole-brain level. These tests were set to a threshold of family-wise error (FWE)–corrected *P* < 0.05. Results are reported in the Supplementary Material.

Based on findings from previous meta-analysis on monetary or social incentive processing in health (Oldham *et al*., 2018; Wilson *et al*., 2018; Gu *et al*., 2019) and relevant neuroimaging research in individuals with depression (Ubl *et al*., 2015), we created masks including ACC (Knutson *et al*., 2008; McCabe *et al*., 2009; Smoski *et al*., 2009; Gotlib *et al*., 2010; Smoski *et al*., 2011; Dichter *et al*., 2012; McCabe *et al*., 2012), insula (Silk *et al*., 2014; Kumar *et al*., 2017; Jankowski *et al*., 2018), striatum (caudate and putamen; Forbes, 2009; Kohls *et al*., 2013; McCabe *et al*., 2009; Pizzagalli *et al*., 2009; Smoski *et al*., 2009; Stoy *et al*., 2012) and VLPFC (Silk *et al*., 2014; Kumar *et al*., 2017; Jankowski *et al*., 2018) to perform the region of interest (ROI) analyses. ROIs specified by masks were derived from the automated anatomical labelling in the Wake Forest University Pick Atlas (WFU Pick Atlas v2.5; http://fmri.wfubmc.edu/software/PickAtlas). Peak activations were extracted from ROIs that reached significance threshold of *P* < 0.012 with false discovery rate (FDR) correction (Bonferroni adjusted accounting for four ROIs) using the MarsBaR function (Matthew *et al*., 2002). Maximum peak activations were then compared using follow-up group-by-condition ANOVA with task (MID, SID) and valence (gain, loss) as within- and group (individuals with SD, HCs) as the between-subject factor, unless otherwise stated.

#### Brain-behavioural correlations

For each participant, beta weights were extracted from ROIs that reached significance during second-level analyses for both anticipation and consumption stages. The extracted beta weights represent the magnitude of peak activation for each significant ROI, which was used to test for the degree of association between self-reported measures and anticipation- and consumption-related brain activations.

#### Task-dependent functional connectivity analysis

To further explore the group differences in functional connectivity, we used a generalized form of task-dependent psychophysiological interaction (gPPI, http://www.nitrc.org/projects/gppi) to examine task-dependent functional connectivity of brain regions associated with both anticipation and consumption of social feedback to shed light on the neural networks involved (McLaren *et al*., 2012). The peak voxel of ROIs that reached significance during second-level analyses were used to create volumes of interest (VOIs) for each participant. Specifically, for each participant, a VOI was generated by creating a 6 mm sphere around this voxel (Ford and Kensinger, 2014). The first eigenvariate was extracted from the time series of the voxels in the specific clusters for each participant. Then, the extracted time series were deconvolved so as to uncover neuronal activity (i.e. physiological variable). The resulting time series were then multiplied with the task design vector and reconvolved with the canonical HRF to form the PPI regressor of interest. These regressors along with the task conditions and the eigenvariate time course were all included in the first-level GLM. Contrast images were then entered into a second-level statistical analysis with a two-sample *t*-test. Multiple comparison correction was performed as described for the GLM analysis. All the data and code used in this study could be available via email zhangdd05@gmail.com (D.Z.).

### Statistics

Descriptive data are presented as means ± SEM, unless otherwise stated.

#### Behavioural data

Three-way repeated-measure ANOVA was performed on reaction time (RT) for hits, with task (MID, SID) and valence (gain, loss, control) as the within factors and group (individuals with SD, HCs) as the between factors. Post hoc Bonferroni tests were used to examine pairwise differences in the ANOVA.

#### Neuroimaging data

For ROI analysis, maximum peak activations that reached Bonferroni-corrected significance during second-level analyses were then compared using follow-up group-by-condition ANOVA with task and valence (gain, loss) as within- and group as the between-subject factor, unless otherwise stated.

To assess brain-behavioural correlations, we calculated group-wise Pearson’s partial correlation coefficients separately for individuals with SD and HCs to allow for statistical non-independence (*P* < 0.05; two-tailed; Poldrack and Mumford, 2009; Ubl *et al*., 2015). Depression severity measured by the SDS was controlled. The Fisher *r*-to-*z* transformation (Zar, 1996) was applied to assess the significance of the difference between two correlation coefficients.

For functional connectivity, independent samples *t*-test was performed (two-tailed) on the extracted gPPI parameter estimates of connectivity between groups.

## Results

### Behavioural results

A significant main effect of valence was found [*F*(2,78) = 11.79, *P* < 0.001, ${\eta}_{\mathrm{p}}^2$= 0.232] with faster RTs in gain (245.32 ± 5.65 ms) and loss conditions (245.37 ± 5.53 ms) than in the control condition (250.75 ± 6.01 ms). No significant group differences were found.

### Whole-brain analyses

#### Within-group analyses

The anticipation and consumption of both incentives were associated with significant response in brain regions including all ROIs, i.e. ACC, striatum, insula and VLPFC, which are known to be responsible for the processing of reward and loss. This was observed in both HC and SD groups (Supplementary Tables S1 and S2).

#### Between-group analyses

No regions showed between-group differences surviving correction at *P* < 0.05 (FWE-corrected) in whole-brain analysis. In the following sections, we report results for the between group comparisons using the previously defined ROIs (Table 2).

**Table 2:** Result of the ROI analysis. Data are thresholded at *P* < 0.012 (Bonferroni-adjusted FDR), with MNI coordinates listed. R: right. L: left

Region	Cluster size, voxels	*z* score	*P* value	*x*	*y*	*z*
**Anticipation of social loss *vs* social control** **Individuals with SD > HCs**						
L ACC	11	3.97	0.010	0	21	−9
**Consumption of social gain *vs* social control** **Individuals with SD < HCs**						
L putamen	13	3.89	0.011	−21	3	12
**Consumption of social loss *vs* social control** **Individuals with SD < HCs**						
L insula	16	4.18	0.008	−36	6	−9

### Neural activation in ROIs

Statistical results of ANOVAs are listed in Table 3.

**Table 3:** Significant results of the ANOVAs for neural activation in ROIs

Effects	ACC	Putamen	Insula
Group	*F*(1,39) = 6.96, *P* = 0.012, ${\eta}_{\mathrm{p}}^2$ = 0.151	Not significant	Not significant
Task	Not significant	Not significant	Not significant
Valance	Not significant	Not significant	*F*(1,39) = 5.52, *P* = 0.024, ${\eta}_{\mathrm{p}}^2$= 0.124
Group × task	*F*(1,39) = 6.27, *P* = 0.017, ${\eta}_{\mathrm{p}}^2$ = 0.138	*F*(1,39) = 11.70, *P* = 0.001, ${\eta}_{\mathrm{p}}^2$ = 0.231	*F*(1,39) = 18.38, *P* < 0.001, ${\eta}_{\mathrm{p}}^2$= 0.320
Group × valance	*F*(1,39) = 4.16, *P* = 0.048, ${\eta}_{\mathrm{p}}^2$ = 0.096	Not significant	Not significant
Task × valance	Not significant	Not significant	Not significant
Group × task × valance	Not significant	Not significant	Not significant

#### Anticipation

**Fig. 2:**
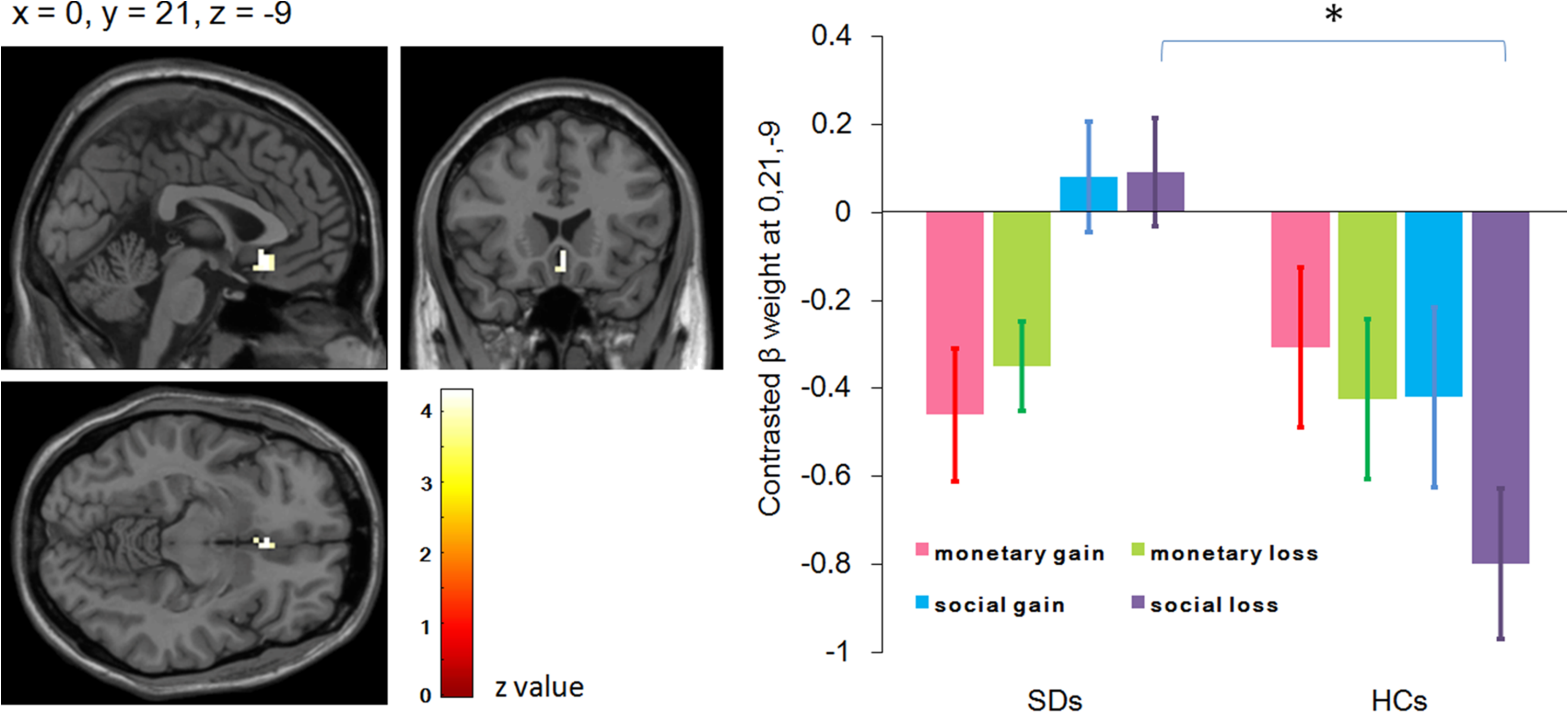
Activation foci showing increased activity in individuals with subthreshold depression (SD) compared with healthy controls (HCs) during anticipation of social loss *vs* social control and the follow-up interaction effect between task and group in the left subgenual anterior cingulate cortex (ROI analysis, *P* < 0.012, Bonferroni-adjusted FDR, displayed on the SPM canonical template). All conditions (monetary gain, monetary loss, social gain and social loss) have been contrasted by relevant control conditions.

Group contrasts for social loss *vs* social control revealed significantly increased activity in SDs compared to HCs in the left sgACC (*x* = 0, *y* = 21, *z* = −9; *P* = 0.01, *z* = 3.97; Figure 2). The ANOVA revealed a main effect of group: SDs (−0.16 ± 0.09) showed enhanced sgACC response compared to HCs (−0.49 ± 0.09). A significant interaction was observed between group and task: while activation in SDs and HCs did not differ in the MID task [*F*(1,39) < 1; SDs = −0.41 ± 0.13, HCs = −0.37 ± 0.13]; SDs (0.09 ± 0.14) showed enhanced response compared to HCs in the SID task [−0.61 ± 0.14; *F*(1,39) = 12.31, *P* = 0.001]. Furthermore, while no significant activation difference was found in HCs between the MID and SID tasks [*F*(1,39) = 1.35, *P* = 0.253; MID = −0.37 ± 0.13, SID = −0.61 ± 0.14], SDs showed enhanced response in the SID task (0.09 ± 0.14) compared to the MID task [−0.41 ± 0.13; *F*(1,39) = 5.73, *P* = 0.022]. The interaction between group and valence was also significant. While SDs and HCs did not differ for the gain condition [*F*(1,39) = 1.48, *P* = 0.231; SDs = −0.19 ± 0.10, HCs = −0.36 ± 0.10], SDs (−0.13 ± 0.10) showed enhanced response compared to HCs [−0.61 ± 0.11] in the loss condition [*F*(1,39) = 10.58, *P* = 0.002]. Furthermore, while SDs did not show significantly different activation in gain compared to loss conditions [*F*(1,39) < 1; gain = −0.19 ± 0.10, loss = −0.13 ± 0.10], HCs exhibited enhanced response in the gain condition (−0.36 ± 0.10) compared to the loss condition [−0.61 ± 0.11; *F*(1,39) = 5.24, *P* = 0.028; Figure 2).

#### Consumption

**Fig. 3:**
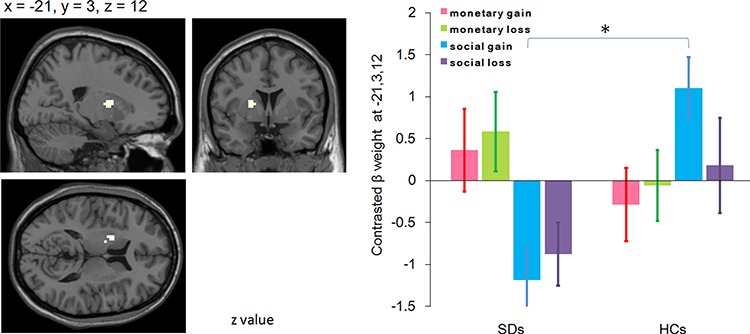
Activation foci showing decreased activity in individuals with SD compared with HCs during consumption of social gain *vs* social control and the follow-up interaction effect between task and group in the left putamen (ROI analysis, *P* < 0.012, Bonferroni-adjusted FDR). All conditions have been contrasted by relevant control conditions.

Group contrasts for social gain *vs* social control revealed significantly decreased activity in SDs compared with HCs in the left striatum (putamen; *x* = −21, *y* = 3, *z* = 12; *P* = 0.011, *z* = 3.89; Figure 3). The ANOVA revealed a significant interaction between group and task: while activation in SDs and HCs did not differ in the MID task [*F*(1, 39) = 1.34, *P* = 0.254; SDs = 0.47 ± 0.39, HCs = −0.17 ± 0.40], SDs (−1.03 ± 0.36) showed reduced response compared to HCs in the SID task [0.64 ± 0.37; *F*(1,39) = 10.66, *P* = 0.002]. Furthermore, while no significant activation difference was found in HCs between the MID task and SID task [*F*(1,39) = 2.83, *P* = 0.101; MID = −0.17 ± 0.40, SID = 0.64 ± 0.37], SDs showed reduced response in the SID task (−1.03 ± 0.36) compared to that in the MID task [0.47 ± 0.39; *F*(1,39) = 10.08, *P* = 0.003; Figure 3).

**Fig. 4:**
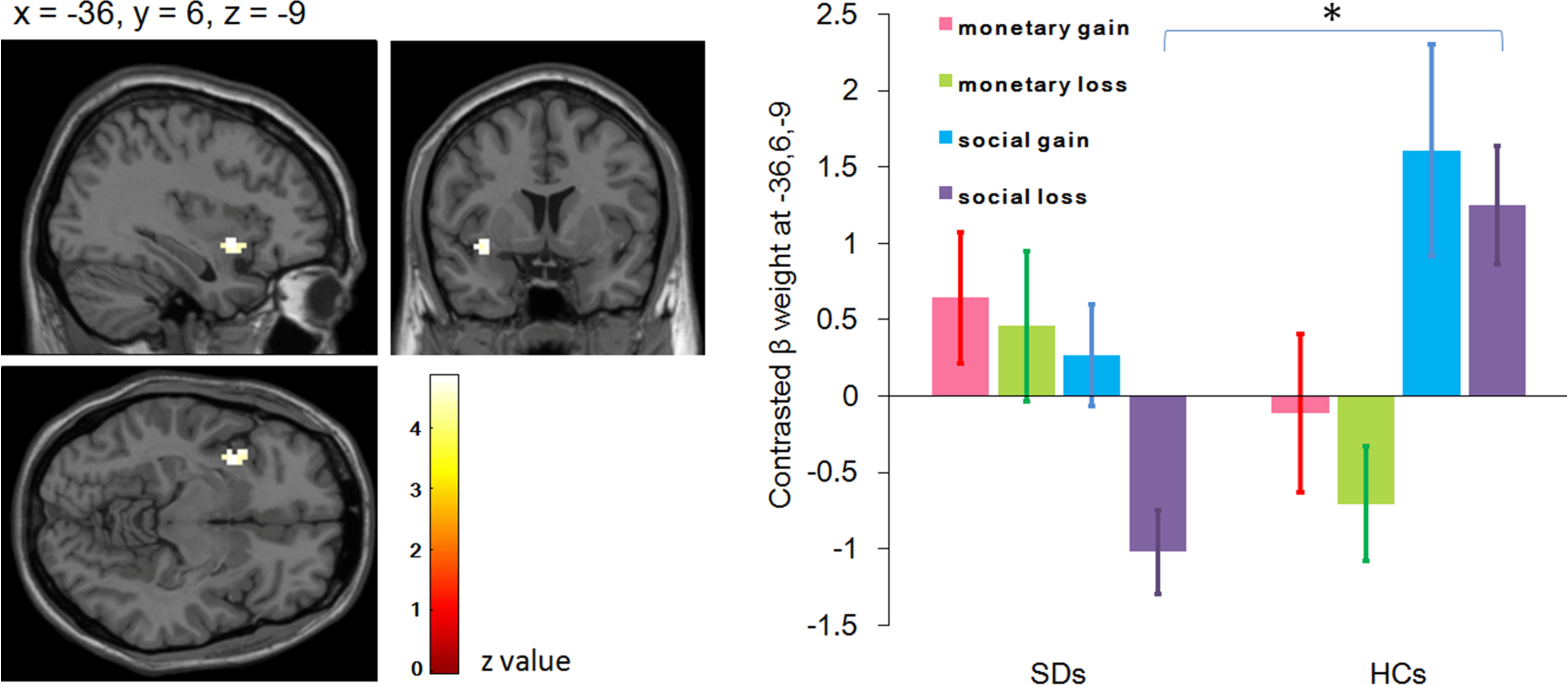
Activation foci showing decreased activity in individuals with SD compared with HCs during consumption of social loss *vs* social control and the follow-up interaction effect between task and group in the left insula (ROI analysis, *P* < 0.012, Bonferroni-adjusted FDR). All conditions have been contrasted by relevant control conditions.

Group contrasts for social loss *vs* social control revealed significantly decreased activity in SDs compared to HCs in the left anterior insula (*x* = −36, *y* = 6, *z* = −9; *P* = 0.008, *z* = 4.18; Figure 4). The ANOVA revealed a significant main effect of valence, with enhanced response in the gain condition (0.60 ± 0.30) compared to that in the loss condition (−0.01 ± 0.17). The interaction between group and task was also significant: while activation in SDs and HCs did not differ in the MID task [*F*(1,39) = 3.09, *P* = 0.09; SDs = 0.55 ± 0.38, HCs = −0.40 ± 0.39], SDs (−0.37 ± 0.34) showed reduced response compared to HCs in the SID task [1.43 ± 0.35; *F*(1,39) = 13.90, *P* = 0.001]. Furthermore, while HCs showed enhanced response in the SID task (1.43 ± 0.35) compared to that in the MID task [−0.40 ± 0.39; *F*(1,39) = 15.85, *P* < 0.001], SDs showed reduced response in the SID task (−0.37 ± 0.34) compared to that in the MID task [0.55 ± 0.38; *F*(1,39) = 4.24, *P* = 0.046; Figure 4).

#### Brain-behavioural correlations

**Fig. 5:**
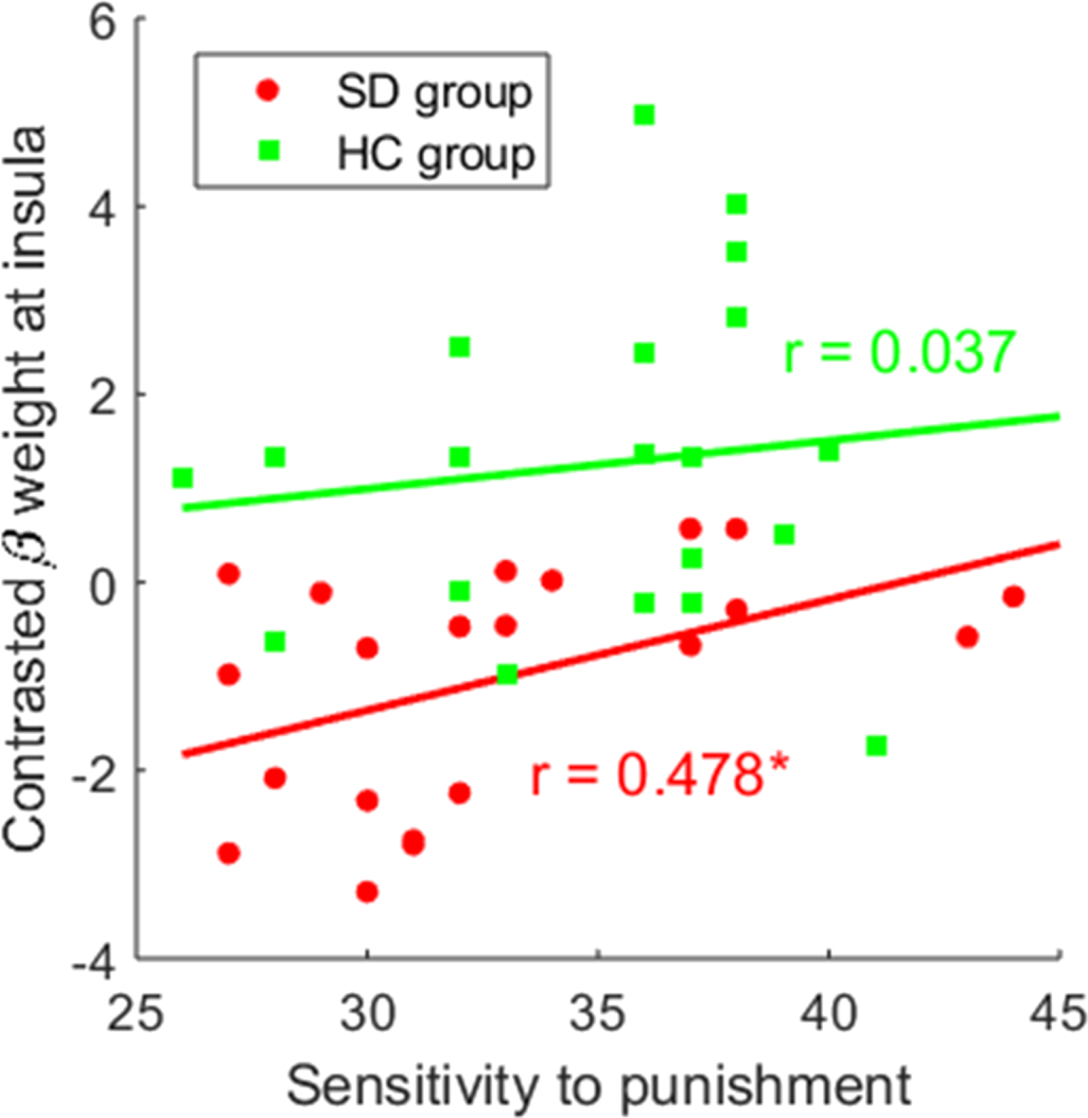
Scatter plot of partial correlations between the sensitivity to punishment (assessed by SPSRQ) and the maximal peak activation (beta weights) at insula during consumption of social loss *vs* social control. The correlation was adjusted for depression severity measured by the SDS scale.

Partial correlation was performed between individual beta weights of statistically significant ROIs and self-reported measures. Result revealed that sensitivity to punishment (measured by the SPSRQ) was positively correlated with insula activity during the consumption of social loss (*r*_part_ = 0.478, df = 18, *P* = 0.033) in SDs (Figure 5). No significant correlations were found in HCs (*r*_part_ = 0.037, df = 17, *P* = 0.880). Fisher *r*-to-*z* transformation revealed no significant difference of those two correlation coefficients between SDs and HCs (*z* = 1.43, *P* = 0.153).

#### Functional connectivity

**Fig. 6:**
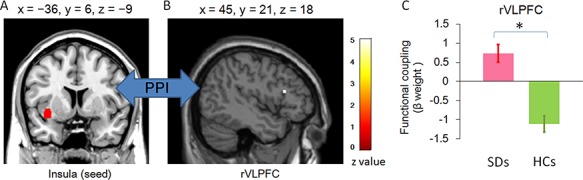
Functional connectivity between insula and rVLPFC during the consumption of social loss *vs* social control. (A) Coronal view of insula (seed), defined by peak voxel that reached significance during second-level analyses. Then, a VOI was generated by creating a 6 mm sphere around this voxel. (B) Sagittal view of rVLPFC showing increased functional connectivity in individuals with SD. (C) The bar chart of the parameter estimates and SEM in the rVLPFC cluster.

The connectivity between the left insula (*x* = −36, *y* = 6, *z* = −9) and right VLPFC (*x* = 45, *y* = 21, *z* = 18; *z* = 4.87, κ = 1) was significantly larger in SDs (0.73 ± 0.24) than in HCs (−1.12 ± 0.21; *t*(39) = 5.807, *P* < 0.001) for the contrast of social loss *vs* social control in the consumption stage (Figure 6). Specifically, SDs showed a positive, whereas HCs showed a negative connectivity between the two regions.

## Discussion

The current study aimed to disentangle neural engagement of anticipating and consuming monetary *vs* social incentives in individuals with subthreshold depression. Results showed that, relative to HCs, individuals with SD showed elevated sgACC activity for anticipation of social loss, reduced putamen activity for consumption of social gain, and diminished insula response during consumption of social loss. In contrast, no group differences were observed for monetary incentive processing. The results therefore indicate specific neural dysfunction-associated social, but not non-social, incentive processing in individuals with SD.

Behavioural performance in individuals with SD and HCs was similar: both groups responded faster on reward and loss trials than on no-incentive trials, regardless of the incentive types. This result suggests that valence manipulations in both tasks successfully modified motivation to respond. The lack of group differences in behavioural performance has been reported previously using similar tasks in individuals with clinical (Knutson *et al*., 2008; Pizzagalli *et al*., 2009; Smoski *et al*., 2009; Stoy *et al*., 2012; Ubl *et al*., 2015) and subclinical depression (Mori *et al*., 2016). A possible reason is that the MID task is more sensitive to neural response than to behavioural performance (Mori *et al*., 2016). This explanation may also apply to the similar SID task.

In this study, individuals with SD exhibited enhanced responses in sgACC compared to HCs during social loss anticipation. BOLD signals in sgACC, a region strongly linked to both social pain (Eisenberger, 2012; Rotge *et al*., 2015) and an aetiology of depression (Drevets *et al*., 2008), have been shown to be elevated during social exclusion in depressed individuals (Silk *et al*., 2014) and could predict increases in depressive symptoms (Masten *et al*., 2011). The sgACC has been suggested to play a role not only in generation and recognition (Phillips *et al*., 2003) but also in anticipation of negative emotion (Ploghaus *et al*., 2003; Nitschke *et al*., 2006; Straube *et al*., 2009). We here demonstrated that individuals with SD over-recruited sgACC during anticipation of social loss, which is consistent with hyper-responsivity of the sgACC to social loss as suggested in previous research.

Relative to controls, individuals with SD exhibited weaker left putamen response as they consumed social gain. Decreased putamen response during the consumption of reward has been found in individuals at high risk of depression (Gotlib *et al*., 2010), MDD participants (Knutson *et al*., 2008), and those with remitted depression (McCabe *et al*., 2009), indicating that it could represent a potential endophenotypic marker for depression. The putamen is an important striatal structure for both money and social reward (Izuma *et al*., 2008) and has been shown to be related to anhedonia in depression (Keedwell *et al*., 2005). However, stimuli used in previous studies have to date been restricted to monetary gain, food reward or happy facial expressions with no or limited social approval meaning. Our finding corroborates and extends prior research by suggesting that, similar to non-social rewards (food or money), social rewards (i.e. positive social feedback) elicit reduced activation within the putamen in depression. This finding is also in agreement with the notion that hypoactivation of striatal areas could be an underlying mechanism for social anhedonia in depression (Kupferberg *et al*., 2016). However, it should be noted that putamen activity was not correlated with self-reported social anhedonia in our study. More studies are needed to further examine the issue using other paradigms.

We also found decreased left insula activation in response to social loss outcome in individuals with SD compared to HCs. This finding is consistent with a previous study, which observed lower insula activation in response to social evaluative threat in a subclinical depression group compared to a healthy group (Dedovic *et al*., 2014). The finding is also in accord with previous studies using negative non-social emotional stimuli, which showed reduced insula activity in people with current (Davidson *et al*., 2003; Kumari *et al*., 2003; Lee *et al*., 2007) and remitted (Thomas *et al*., 2011) depression. A meta-analysis of depression has also highlighted the hypoactivated insula in resting state paradigms and negative emotion induction studies (Fitzgerald *et al*., 2008). However, an opposite pattern of insula activation (hyperactivation) has been reported in response to social rejection in currently depressed youth (Silk *et al*., 2014; Jankowski *et al*., 2018) and older adult patients (Kumar *et al*., 2017). A possible explanation for the inconsistency in social-related studies is that different samples of depressed individuals were used. Those with subclinical depression may differ in their insula response (hypoactivity) to negative social stimuli compared to those with clinical depression (hyperactivity). Although the findings of the social rejection studies on clinical depression differ from the current study, probably because of the nature of the population recruited or the tasks employed, our findings do suggest that abnormalities in the neural substrate of social loss might be present in subclinical individuals before the onset of MDD. Future research could untangle this issue by comparing both subclinical and clinical samples.

Insula is known to be activated by aversive stimuli (e.g. social loss in the current study; Caria *et al*., 2010). Decreased insula activation in individuals with SD may indicate reduced negative emotional response to social loss. It is therefore worth considering whether there is a group difference of the underpinning neural networks associated with insula. Therefore, this study performed a PPI analysis to examine changes in functional connectivity between the insula and other ROIs. In addition to the above reason, we selected this region as a seed for the PPI analysis because of its modulation by positive and negative emotions (Uddin, 2015), especially social stress (exclusion; Wang *et al*., 2017), as well as its implicated role in both affective and social functioning (Lamm and Singer, 2010; Kumar *et al*., 2017). We discovered an increased insula functional connectivity with rVLPFC during social loss consumption in individuals with SD. Specifically, this coupling is positive in individuals with SD, while it is negative in HCs. Converging evidence in healthy participants has revealed that both are key regions in processing of ‘social pain’, and VLPFC negatively correlates with activities in the insula, reflecting a function of emotion regulation (Eisenberger *et al*., 2003; Lorenz *et al*., 2003; Masten *et al*., 2009; Wang *et al*., 2017). Our finding in HCs is in line with these previous accounts. However, positive VLPFC-insula functional activity in individuals with SD might reveal a failure on the part of the pre-frontal area to suppress insula functioning. Adopting a broader neural network view, dysfunctional cortical–limbic circuitry has been well-reported in individuals with depression (Seminowicz *et al*., 2004; Anand *et al*., 2005; Langenecker *et al*., 2007). Aberrant function in insula/VLPFC, as the key regions in this network (Sliz and Hayley, 2012), has been shown to be responsible for impaired emotion salience processing and maladaptive emotion regulation and thus leading to depressogenic information processing deficits (Jankowski *et al*., 2018). In the current study, the abnormally strengthened connectivity between the two areas may cause insensitivity to and insufficient regulation of these brief social challenges. Therefore, our results extend previous studies by indicating that dysfunctional cortical–limbic circuitry are not only involved in basic emotion processing (Anand *et al*., 2005) but also implicated in the processing of social signals. In individuals with SD, greater sensitivity to punishment as assessed by the SPSRQ scale was found to be associated with increased activation in insula during social loss consumption. This result suggests that individuals with SD who reported higher punishment sensitivity recruited stronger insula response to social loss, which might indicate that depressogenic insula activity was modulated by self-reported punishment sensitivity.

No differences were observed between individuals with SD and HCs in reward network activation during the monetary incentive processing. These findings are counter to previous research using the MID task, which found altered fronto-cingulate-striatal responses in clinical depression (Knutson *et al*., 2008; Pizzagalli *et al*., 2009; Zhang *et al*., 2013). Again, this may relate to studying subclinical participants in the present study, compared to findings in those with clinical depression in previous studies. To our knowledge, only one study has used this task in subclinical depression: Mori *et al*. (2016) showed hypoactive responsiveness in the left VLPFC and angular gyrus during a MID task in individuals with SD, which can be normalized by psychotherapy. However, neural alterations observed in their study were not located in typical monetary reward-related regions and, as the authors suggest, their sample size is relatively small (*n* = 15). Therefore, it is possible that subclinical populations with depression show reward-processing abnormalities that are specific to social incentives, while those with clinical symptoms show more widespread reward-related abnormalities extending to non-social domains.

Several limitations should be noted. First, we did not obtain a direct self-report comparison of the pleasantness or averseness of monetary *vs* social incentives. However, behavioural results found that gain and loss trials were associated with faster RTs than neutral trials for both monetary and social runs, indicating both may function in a similar way to change behaviour. Second, the monetary and social incentives used in this study are both secondary incentives, i.e. no inherent reward or loss in itself and for which incentive value must be learned (Rizvi *et al*., 2016). We did not include a comparison with a primary incentive (e.g. food). There is evidence for different neural mechanisms between the processing of primary and secondary rewards (Sescousse *et al*., 2013), and hedonic function has been shown to be disrupted in depression using food rewards as stimuli (Rizvi *et al*., 2016). It would be interesting if future studies on subclinical and clinical depression can probe the processing of primary *vs* secondary incentives. Third, most of the subjects in SD group only had mild depression symptoms (mean SDS sore of 0.54 and mean BDI-II score of 16), which might result in false negative findings in this study. Lastly, our study was carried out exclusively in young adults. These findings may therefore not be representative of those seen in older adults. However, it should be noted that the highest prevalence of major depressive disorder is seen in those aged 20–29 years (Ferrari *et al*., 2013). Understanding the biological and psychological factors that pre-dispose people to initial episodes of clinical depression is therefore a key to effective, early interventions. Furthermore, in older cohorts, multiple episodes, chronic symptoms and effects of treatment (pharmacological or psychological) may represent confounds.

In sum, our findings provide evidence that SD is characterised by distinct neural alteration in processing monetary and social incentives. The two incentives evoked distinct patterns of neural alteration in sgACC, putamen, insula and rVLPFC, which are specific to social incentive processing and largely reflect ‘social pain’ network dysfunction. Although there are some discrepancies between our findings and previous work, this could be at least partly explained by clinical status and demographic differences in different population samples. Our finding suggests that social incentive processing abnormalities may be more trait-like than non-social reward. This research may help us understand how specific brain dysfunctions in depression may contribute to their deficits in processing social incentives.

## Funding

This study was funded by the National Natural Science Foundation of China (31571120), Shenzhen Basic Research Project (JCYJ20180305124305294; JCYJ20150729104249783) and Guangdong-Government Funding for Scientific Research (2015KCXTD009, 2016KZDXM009).

## Supplementary Material

scan-19-128-File008_nsz061
