# Supplementary material for: Neural substrates for anticipation and consumption of social and monetary incentives in depression
Source: Soc Cogn Affect Neurosci. 2019 Sep 11;14(8):815–26. doi: 10.1093/scan/nsz061 (PMC6847340; doi:10.1093/scan/nsz061)
Supplement: scan-19-128-File008_nsz061 [file scan-19-128-file008_nsz061.docx]

Table S1. Within-group results of the whole-brain analysis in healthy controls (n = 20). Data are thresholded at *p* < 0.05 (FWE-corrected), with MNI coordinates listed. R: right. L: left.

| Region | Cluster size,  Voxels | z score | p value | x | y | z |
| --- | --- | --- | --- | --- | --- | --- |
| **Anticipation of monetary gain > monetary control** | | | | | | |
| *R caudate* | 5 | 5.03 | 0.014 | 9 | 2 | 12 |
| L inferior occipital cortex | 3 | 5.00 | 0.017 | 33 | −81 | −9 |
| **Anticipation of monetary loss > monetary control** | | | | | | |
| *L caudate* | 3 | 4.86 | 0.035 | −12 | 24 | −6 |
| **Anticipation of monetary loss < monetary control** | | | | | | |
| R MPFC | 2 | 4.84 | 0.039 | 12 | 27 | 57 |
| **Anticipation of social loss < social control** | | | | | | |
| *L ACC* | 27 | 5.69 | <0.001 | −3 | 48 | 9 |
| L MPFC | 2 | 4.83 | 0.040 | 0 | 54 | 0 |
| **Anticipation of (monetary and social) loss < control** | | | | | | |
| L MPFC | 7 | 5.17 | 0.007 | 0 | 54 | 0 |
| **Consumption of monetary gain > monetary control** | | | | | | |
| L inferior occipital cortex | 95 | 5.84 | <0.001 | −21 | −90 | −12 |
| L inferior occipital cortex |  | 5.73 | <0.001 | −24 | −96 | −6 |
| R lingual gyrus | 73 | 5.83 | <0.001 | 24 | −90 | −3 |
| R inferior occipital cortex |  | 5.62 | 0.001 | 30 | −96 | −3 |
| *R putamen* | 6 | 5.03 | 0.013 | 21 | 6 | 12 |
| L fusiform | 4 | 4.90 | 0.028 | −36 | −45 | −18 |
| **Consumption of monetary loss > monetary control** | | | | | | |
| L cerebellum_crus2 | 3 | 4.98 | 0.018 | 0 | −84 | −21 |
| **Consumption of social gain > social control** | | | | | | |
| *L putamen* | 18 | 5.02 | 0.015 | −24 | 5 | 16 |
| L fusiform | 2 | 4.88 | 0.032 | −33 | −45 | −24 |
| **Consumption of social loss > social control** | | | | | | |
| L middle occipital cortex | 16 | 5.50 | 0.001 | −27 | −99 | −3 |
| *L insula* | 12 | 5.47 | 0.013 | −39 | 3 | 2 |
| **Consumption of social loss < social control** | | | | | | |
| *R VLPFC* | 7 | 4.95 | 0.022 | 45 | 12 | 27 |
| **Consumption of (monetary and social) gain> control** | | | | | | |
| R inferior occipital cortex | 39 | 5.49 | 0.001 | 21 | −93 | −6 |
| L middle occipital cortex | 18 | 5.02 | 0.015 | −27 | −99 | −3 |
| L inferior occipital cortex |  | 4.93 | 0.024 | −30 | −96 | −12 |
| L inferior occipital cortex |  | 4.86 | 0.035 | −21 | −90 | −9 |
| L fusiform | 2 | 4.88 | 0.032 | −33 | −45 | −24 |
| R fusiform | 2 | 4.79 | 0.049 | 39 | −66 | −21 |

Regions written in italic type denote task-specific responses matching hypothesis-driven regions of interest.

Table S2. Within-group results of the whole-brain analysis in individuals with depression (n = 21). Data are thresholded at *p* < 0.05 (FWE-corrected).

| Region | Cluster size,  Voxels | z score | p value | x | y | z |
| --- | --- | --- | --- | --- | --- | --- |
| **Anticipation of monetary gain > monetary control** | | | | | | |
| *R caudate* | 3 | 4.88 | 0.030 | 8 | 6 | 9 |
| L fusiform | 2 | 4.87 | 0.033 | −36 | −60 | −18 |
| L inferior occipital cortex | 2 | 4.84 | 0.038 | −30 | −90 | −9 |
| L inferior occipital cortex | 2 | 4.81 | 0.043 | −21 | −90 | −6 |
| **Anticipation of monetary loss > monetary control** | | | | | | |
| R supplementary motor area | 2 | 5.48 | 0.001 | 15 | −24 | 57 |
| R thalamus | 4 | 5.30 | 0.003 | 21 | −21 | 9 |
| L postcentral gyrus | 5 | 5.00 | 0.017 | −30 | −36 | 63 |
| R DLPFC | 2 | 4.95 | 0.022 | 18 | −15 | 57 |
| *R caudate* | 3 | 4.84 | 0.037 | 9 | 0 | 12 |
| R thalamus | 2 | 4.80 | 0.046 | 9 | −27 | −3 |
| **Anticipation of social loss > social control** | | | | | | |
| *L ACC* | 5 | 5.49 | 0.001 | −2 | 30 | −6 |
| **Anticipation of (monetary and social) gain > control** | | | | | | |
| L fusiform | 2 | 5.13 | 0.008 | −39 | −63 | −15 |
| L inferior occipital cortex | 2 | 4.97 | 0.020 | −30 | −90 | −9 |
| R inferior occipital cortex | 2 | 4.90 | 0.027 | 33 | −90 | −3 |
| L middle occipital cortex | 2 | 4.85 | 0.036 | −30 | −96 | −6 |
| **Anticipation of (monetary and social) loss > control** | | | | | | |
| *R caudate* | 3 | 4.89 | 0.029 | 9 | 6 | 3 |
| **Consumption of monetary gain > monetary control** | | | | | | |
| R inferior occipital cortex | 191 | 7.27 | <0.001 | 33 | −87 | −6 |
| R calcarine sulcus |  | 5.87 | <0.001 | 24 | −96 | 0 |
| R middle occipital cortex |  | 5.84 | <0.001 | 30 | −90 | 3 |
| L calcarine sulcus | 153 | 6.46 | <0.001 | −18 | −99 | −6 |
| L middle occipital cortex |  | 6.27 | <0.001 | −21 | −90 | 0 |
| L inferior occipital cortex |  | 5.31 | 0.003 | −33 | −87 | −9 |
| R fusiform | 36 | 5.83 | <0.001 | 36 | −54 | −18 |
| L fusiform | 73 | 5.54 | 0.001 | −27 | −60 | −12 |
| L fusiform |  | 5.34 | 0.003 | −30 | −51 | −15 |
| L fusiform |  | 5.34 | 0.003 | −30 | −75 | −12 |
| R inferior temporal cortex | 2 | 4.85 | 0.035 | 48 | −45 | −12 |
| *R putamen* | 3 | 4.83 | 0.039 | 25 | 4 | 9 |
| **Consumption of money loss > money control** | | | | | | |
| L precuneus | 2 | 4.88 | 0.031 | 0 | −60 | 57 |
| **Consumption of social gain > money control** | | | | | | |
| L inferior occipital cortex | 2 | 4.81 | 0.043 | −42 | −84 | −12 |
| *R VLPFC* | 4 | 4.80 | 0.047 | 45 | 9 | 27 |
| **Consumption of social gain < money control** | | | | | | |
| *L putamen* | 19 | 5.50 | 0.001 | −21 | 6 | 11 |
| *L VLPFC* | 5 | 5.01 | 0.016 | −48 | 27 | 24 |
| **Consumption of social loss > money control** | | | | | | |
| L inferior occipital cortex | 28 | 5.48 | 0.001 | −45 | −60 | −12 |
| **Consumption of social loss < money control** | | | | | | |
| *L insula* | 10 | 5.00 | 0.017 | −41 | 6 | −2 |
| *R VLPFC* | 8 | 5.39 | 0.001 | 42 | 12 | 30 |
| **Consumption of (monetary and social) gain > control** | | | | | | |
| R inferior occipital cortex | 165 | 6.64 | <0.001 | 33 | −90 | −6 |
| R middle occipital cortex |  | 5.95 | <0.001 | 27 | −93 | 6 |
| R calcarine sulcus |  | 5.22 | 0.005 | 15 | −96 | −3 |
| L calcarine sulcus | 127 | 6.22 | <0.001 | −18 | −102 | −6 |
| L middle occipital cortex |  | 6.11 | <0.001 | −24 | −93 | 0 |
| L inferior occipital cortex |  | 5.10 | 0.010 | −33 | −87 | −6 |
| L fusiform | 8 | 5.45 | 0.001 | −30 | −75 | −12 |
| L fusiform | 1 | 4.96 | 0.021 | −33 | −84 | −15 |
| L fusiform | 3 | 4.90 | 0.027 | −33 | −51 | −15 |
| R fusiform | 2 | 4.88 | 0.031 | 36 | −57 | −15 |

Regions written in italic type denote task-specific responses matching hypothesis-driven regions of interest.
